# Oral Neutrophil Transcriptome Changes Result in a Pro-Survival Phenotype in Periodontal Diseases

**DOI:** 10.1371/journal.pone.0068983

**Published:** 2013-07-11

**Authors:** Flavia S. Lakschevitz, Guy M. Aboodi, Michael Glogauer

**Affiliations:** 1 Department of Periodontology, Faculty of Dentistry, University of Toronto, Toronto, Ontario, Canada; 2 Matrix Dynamics Group, Faculty of Dentistry, University of Toronto, Toronto, Ontario, Canada; University of Cincinnati, United States of America

## Abstract

**Background:**

Periodontal diseases are inflammatory processes that occur following the influx of neutrophils into the periodontal tissues in response to the subgingival bacterial biofilm. Current literature suggests that while neutrophils are protective and prevent bacterial infections, they also appear to contribute to damage of the periodontal tissues. In the present study we compare the gene expression profile changes in neutrophils as they migrate from the circulation into the oral tissues in patients with chronic periodontits and matched healthy subjects. We hypothesized that oral neutrophils in periodontal disease patients will display a disease specific transcriptome that differs from the oral neutrophil of healthy subjects.

**Methods:**

Venous blood and oral rinse samples were obtained from healthy subjects and chronic periodontitis patients for neutrophil isolation. mRNA was isolated from the neutrophils, and gene expression microarray analysis was completed. Results were confirmed for specific genes of interest by qRT-PCR and Western Blot analysis.

**Results and Discussion:**

Chronic periodontitis patients presented with increased recruitment of neutrophils to the oral cavity. Gene expression analysis revealed differences in the expression levels of genes from several biological pathways. Using hierarchical clustering analysis, we found that the apoptosis network was significantly altered in patients with chronic inflammation in the oral cavity, with up-regulation of pro-survival members of the Bcl-2 family and down-regulation of pro-apoptosis members in the same compartment. Additional functional analysis confirmed that the percentages of viable neutrophils are significantly increased in the oral cavity of chronic periodontitis patients.

**Conclusions:**

Oral neutrophils from patients with periodontal disease displayed an altered transcriptome following migration into the oral tissues. This resulted in a pro-survival neutrophil phenotype in chronic periodontitis patients when compared with healthy subjects, resulting in a longer-lived neutrophil. This is likely to impact the severity and length of the inflammatory response in this oral disease.

## Introduction

Periodontal diseases (PD) are inflammatory conditions involving innate and adaptive immune cells that occur in response to the presence of subgingival bacteria [1], [2]. Their diagnosis is based on clinical parameters that report on tissue destruction, such as clinical attachment loss (CAL), probing depth (PD) bleeding on probing (BOP), plaque index (PI) and dental radiography [3]. Clinical assessment using these measures is time consuming and since it reports on tissue destruction it does not actually tell the clinician if the patient is in an active phase of the disease process [4]. This last point is critical since PD, like most inflammatory diseases, alternates between periods of tissue destruction and periods of inactivity and routine periodontal examination cannot determine the current state at the time of the examination [1], [5].

Current literature implicates neutrophils (polymorphonuclear leukocytes or PMNs) as the main immune cell responsible for PD progression[5]–[7]. In addition to the presence of neutrophils in the inflamed area, these cells may also be dysfunctional in in PD patients [8]. Previous studies from our group have demonstrated that periodontal patients have increased numbers of neutrophils in the oral cavity [9]. Moreover, a number of studies have demonstrated that peripheral blood neutrophils from patients with periodontitis are augmented in their ability to phagocytose and kill bacteria, consequently release significantly more reactive oxygen species (ROS) and neutrophil elastase compared with healthy controls [2], [10], [11]. These findings clearly demonstrate an alteration in neutrophil function in affected patients thus emphasizing the importance of investigating what accounts for the observed alterations in patients with periodontitis.

Microarray analyses allow us to simultaneously investigate the expression of thousands of genes [12],[13]. This approach has been used to identify target genes associated with type I diabetes [14], arthritis [15] and lupus [16]. In addition, a number of studies have attempted to identify inflammatory markers associated with periodontal diseases [17]–[21]. However, a specific marker for active periodontitis has yet to be identified [22], [23]. The complexity of periodontal disease pathogenesis, known to involve bacterial antigens, cytokines, and other pro-inflammatory mediators, that can reach circulation, increase the difficulty of finding relevant biomarkers. By characterizing neutrophil phenotype changes at the site of inflammation we hope to identify changes in gene expression that could be potentially used as markers of disease activity.

## Materials and Methods

### Study Population

Seventy-six participants enrolled in the study all were systemically healthy, non-smokers. Participants were divided into two groups: Control group, of periodontally healthy patients (Healthy), and test group, comprises of patients with generalized chronic periodontal disease (CP). The two groups presented similar male/female ratio, with a mean age of 53±5.09 years for control group and 59±1.77 years for CP group. A documented history of surgical treatment or antimicrobial therapy was noted for all periodontitis patients. Demographic and clinical parameters of the individuals in this study (mean ± SEM) are provided in Table 1.

**Table 1:** List of primer sequences for qRT-PCR.

GeneSymbol^a^	GenBankAccession #	Primers Sequence (5′–3′)
**GAPDH**	NM_001256799	GGAGCGAGATCCCTCCAAAAT
		GGCTGTTGTCATACTTCTCATGG
**LTF**	NM_002343	ATGGTGGTTTCATATACGAGGCA
		CTTTCGGTCCCGTAGACTTCC
**CXCL10**	NM_001565	GTGGCATTCAAGGAGTACCTC
		TGATGGCCTTCGATTCTGGATT
**CCL2**	NM_002982.3	CAGCCAGATGCAATCAATGCC
		TGGAATCCTGAACCCACTTCT
**ELA**	NM_001972	CTCGCGTGTCTTTTCCTCG
		GCCGACATGACGAAGTTGG
**MPO**	NM_000250	TGCTGCCCTTTGACAACCTG
		TGCTCCCGAAGTAAGAGGGT
**TNF**	NM_000594	CCTCTCTCTAATCAGCCCTCTG
		GAGGACCTGGGAGTAGATGAG

a The top sequence is forward primer and the bottom sequence is reverse primer. List of primers used for validation of microarray results with qRT-PCR. Abbreviations: GAPDH - glyceraldehyde-3-phosphate dehydrogenase, LTF - Neutrophil Lactoferrin, CXCL10 - chemokine (C-X-C motif) ligand 10, CCL2 - chemokine (C-C motif) ligand 2, ELA - neutrophil expressed elastase, MPO – Myeloperoxidase, TNF - tumor necrosis factor.

All participants were patients at the Faculty of Dentistry, University of Toronto, Toronto, ON. Participants provided a written informed consent to participate, and the Office of Research Ethics (ORE) at the University of Toronto approved the study (Protocol # 25698).

### Study Design

All participants received a complete intraoral examination and full periodontal charting on the day of samples collection. The following clinical parameters were assessed at six sites per tooth: probing depth (PD), clinical attachment loss (CAL) and bleeding on probing (BOP). One clinician was responsible for all clinical data collection.

Oral rinse samples were collected as previously described [8], [24]. Participants rinsed with 5 mL of isotonic sodium chloride solution 0.9% (Baxter, Toronto, ON, Canada) for 30 seconds before any probing of the gingival tissues or manipulation of the oral tissues was done, in order to avoid initiating gingival bleeding that might interfere with the results. Patients were then asked to expectorate the rinse sample into a sterile 50 mL falcon tube. Participants were asked to repeat this procedure 6 times (for a total of 30 mL) with 3 minutes intervals between each rinse sample.

Peripheral blood samples were also collected as previously described [8], [24]. Participants provided 10 mL non-fasting venous blood samples.

All laboratory procedures were initiated within 2 hours of sample collection and RNA isolation was completed within the same day of sample collection. RNA samples were kept at −80°C until analyzed.

### Neutrophil Quantification

In order to determine the number of neutrophils recruited to the oral cavity in healthy patients and in patients with localized/generalized chronic periodontits, 0.5 ml of patients’ oral rinse sample was aliquoted for oral neutrophil quantification [9]. Samples were fixed with 50 µl paraformaldehyde 37%, and centrifuged in 16,200×g. Pellet was resuspended in 100 µl of PBS. Cells were then subjected to Acridine orange (AO) staining, as previously described [9]. Briefly, 4 µg/ml of AO were added to each concentrated sample, and mixed well with a vortex mixer for 15 sec. After 15 min of light protected incubation at room temperature (RT), samples were counted with a Hemocytometer.

### Isolation of Blood PMN (PMN-B)

Blood neutrophils isolation was completed using gradient assay - 1 step polymorphus (Axis-Shield PoC, Oslo, Norway). Whole blood samples were laid on the top of 1 step polymorphus layer, then centrifuged (527×g/30 min/RT). Neutrophils were collected from the lower of two bands. Cells were washed with Hank’s Balanced Salt Solution, no calcium, no magnesium (HBSS −/−)(Invitrogen, Grand Island, NY) and hypotonic lysis was performed to remove erythrocyte contamination. Cells were re-suspended in 1 mL HBSS −/−. Count was obtained using a Coulter counter. Isolation procedure resulted in 95% cells viability (assessed by Trypan Blue exclusion test) and purity of 98% (assessed by diff-quick staining, Siemens, Deerfield, IL, USA).

### Isolation of Oral PMN (PMN-O)

PMN-O were obtained from the same patients. Oral rinse samples were filtered through a 40 µm, 20 µm, and 11 µm nylon mesh (Millipore, USA) [24]. The filtered samples were then centrifuged (527×g/10 min/4°C). Isolation procedure resulted in 95% cells viability (assessed by Trypan Blue exclusion test) and purity of 98% (assessed by diff-quick staining, Siemens, Deerfield, IL, USA). We obtain 8.81×10^6^±2.46×10^6^ cells in Healthy subjects and 1.70×10^7^±2.39×10^6^ cells in CP patients.

### RNA Isolation from Purified PMN

Total RNA was isolated from both PMN-B and PMN-O, using mirVana isolation kit (Ambion, Austin, TX, USA). Isolation procedure followed manufacturer suggested protocol. Genomic DNA were eliminated by RNase-free DNase I digestion (Qiagen, Mississauga, ON, Canada) during the isolation procedure. Isolated total RNA was stored at −80°C and later analyzed on an Agilent 2100 bioanalyzer using a RNA 6000 picolabchip kit (Agilent technologies, Santa Clara, CA, USA) [24].

### Microarray

Isolated RNA from PMN-O and PMN-B of 4 healthy controls and 4 generalized chronic periodontits patients were measured for gene expression by microarray using the Illumina Human 12WG Expression BeadChip (48,000 gene transcripts). We pre-processed the raw data using lumi R package (http://www.r-project.org). Background correction was done in Beadstudio (Illumina, San Diego, CA, USA). The quantile normalization method implemented in lumi R package was used to normalize the data. We use LIMMA (linear models for microarray data) to identify differentially expressed genes. To compare gene expression in multiple groups, we used an empirical Bayes (EB) approach to assess differentially expressed genes, allowing for multiple testing adjustments [13]. The method does not require the data to be normally distributed. Briefly, it starts by fitting a linear model for each gene in the data, and then EB method is used to moderate the standard errors for estimating the moderated t-statistics for each gene, which shrinks the standard errors towards a common value. The corresponding p-values for the modified t-statistics were adjusted using the multiple testing procedures developed by Benjamini and Hochberg [25], [26]. Later genes were selected controlling for false discovery rate (FDR) at the level of 0.01 and by fold change of 2. Further analyses were carried out using Ingenuity Pathway Analysis (IPA) software (http://www.ingenuity.com) (Ingenuity Systems, Redwood City, CA, USA). Additional analysis were performed using DAVID 6.7 (http://david.abcc.ncifcrf.gov) (the database for annotation, visualization and integrated discovery) bioinformatics resources as described elsewhere [27], [28] and using GOEAST (http://omicslab.genetics.ac.cn/GOEAST/) (Gene Ontology Enrichment Analysis Software Toolkit) bioinformatics tools [29].

### Generating a Heat Map

We upload text format files to MultiExperiment Viewer (http://www.tm4.org/mev/), and a heat map was produced, to compare neutrophils from blood and oral samples between healthy and chronic periodontits patients [30].

### Accession Codes

The microarray data complies with MIAME guidelines, and the data set was deposited at Gene Expression Omnibus (National Center for Biotechnology Information), accession number: GSE 435525.

### qRT-PCR

Selected genes of interest, generated from microarray analysis, were analyzed using quantitative RT-PCR (qRT-PCR). RNA samples analysed by qRT-PCR were the same RNA samples used for microarray analysis (4 healthy controls, 4 CP patients). The first set of selected genes were chosen because of their importance as known regulators of neutrophil anti-bacterial functions and have also been shown to have altered regulation during inflammatory responses [myeloperoxidase (MPO), neutrophil lactoferrin (LTF), neutrophil expressed elastase (ELA)] [2], [5]. The second set of genes is known to be associated with the overall inflammatory response, yet not neutrophil specific [chemokine (C-X-C motif) ligand 10 (CXCL10), chemokine (C-C motif) ligand 2 (CCL2), tumor necrosis factor (TNF)] [4], [16]. qRT-PCR was performed in triplicates using CFX96TM Real-Time System (Bio-Rad, Hercules, CA, USA). The protocol was followed as previously described [24]. Total RNA (0.12 µg) was reverse transcribed into cDNA using Superscript II (Invitrogen Life Technologies, Burlington, ON, Canada) and Oligo-dT_18_VN primer in a 20 µL reaction system. Two negative controls were used to ensure there was no contaminating DNA: one without template RNA, and another lacking the reverse transcriptase. A reaction mixture was also prepared containing: 5 µL of template cDNA, 15 µL of master mix [1 µL of forward and 1 µL of reverse primer (both 10 µM stock), 10 µL of BioRad Ssofast EvaGreen Super Mix and 3 µL of RNase-free distilled water was used. Primers were designed from the PrimerBank ID number (ACGT Corp., Toronto, ON, Canada) and glyceraldehyde-3-phosphate dehydrogenase (GAPDH) was used to normalize the expression data. Primers used in this study are listed in Table 2.

**Table 2:** Demographics and clinical parameters of study subjects.

	Mean ± SEM	
	Healthy subjects (n = 14)	Generalized Periodontitis (n = 62)
Age (years)	53±5.09	59±1.77*
% of Males	50	54 ^ns^
Pocket depth (mm)		
% of sites with ≥4 mm	1.73±0.81	24.15±2.49*
% of sites with ≥5 mm	0±0	11.99±1.79*
% of sites with ≥6 mm	0±0	5.2±0.95*
Bleeding on Probing	5.38±0.40	32.1±3.52*
% of Active inflammation	0	16

ns - not significant;

* p-value ≤0.05 vs. Healthy subjects.

### Quantification of Neutrophil Apoptosis

Neutrophil apoptosis was quantified in PMN-B and PMN-O samples obtained from CP patients utilizing flow cytometry method as a percentage of cells stained with Annexin V - FITC and Propidium Iodide (PI) using a Annexin-V-Fluos Staining kit (Roche, Mississauga, ON) according to the manufacturer’s instructions and as described elsewhere [31]. Cells stained positive for FITC and negative for PI were considered apoptotic and cells positive for both PI and FITC, and PI only were considered necrotic. Data was analyzed with FlowJo software.

### Western Blot

In order to confirm expression of the proteins identified by the microarray and qRT-PCR analyses, the Western blot technique was used. Genes with altered regulation in the apoptosis pathway were selected in order to validate the data at the protein level. The following proteins were analyzed: B-cell lymphoma-extra large (Bcl- xl), Bcl-2-like protein 11 [Bim (C34C5)], Bcl-2–associated X protein [Bax (D2E11)]. All antibodies were purchased from Cell Signaling Technology (Danvers, MA), including rabbit mAb and anti-rabbit Ig, horseradish peroxidase (HRP)- conjugated antibody. Primary antibodies were used at a 1∶1000 dilution while secondary antibodies were used at 1∶2000 dilution.

Protein lysates from PMN-B and PMN-O were prepared with SDS buffer. The lysates were cleared with 5 minutes centrifugation at 13,000 rpm at 4°C. Total protein concentration was measured with a BCA protein assay kit (Pierce). Fifteen micrograms of the samples of heat-denatured protein was loaded on a 12% polyacrylamide gel. After electrophoresis, the gels were transferred to nitrocellulose filters (Amersham-GE, Baie d’Urfe QC) by electroblotting. After transfer, the filter was incubated for 1 h in a blocking buffer [5% nonfat milk powder in Tris-Buffered Saline and Tween (TBST)]. The membrane was maintained overnight in primary antibody (listed above) (1∶1000) in TBST with 5% Bovine serum albumin (BSA), washed 3×10 min with TBST, and incubated for 60 min in horseradish peroxidase (HRP)- conjugated secondary antibody/5% nonfat milk powder/TBST (1∶2000) at room temperature, following manufacture’s instructions. After 3×10 min washing with TBST, the membrane was developed with Western Lightning solution (Perkin Elmer), and the resulting chemiluminescence was exposed to film (Kodak). The filters were quantitated using ImageJ software and normalized to b β-actin expression, as described in Wang et al, 2013 [32].

### Statistical Analysis

For experiments in which there were multiple observations per sample, numerical results were expressed as mean ± SEM. All experiments were performed at least three times, and within each experiment, each data point had a sample size of n ≥3. Statistical analysis was performed using Student’s two-tailed t test, unless specified otherwise. P≤0.05 was considered statistically significant, by GraphPad analysis (GraphPad software).

## Results

### Oral Chronic Inflammation Increases Neutrophil Recruitment to the Site of Infection

**Figure 1:**
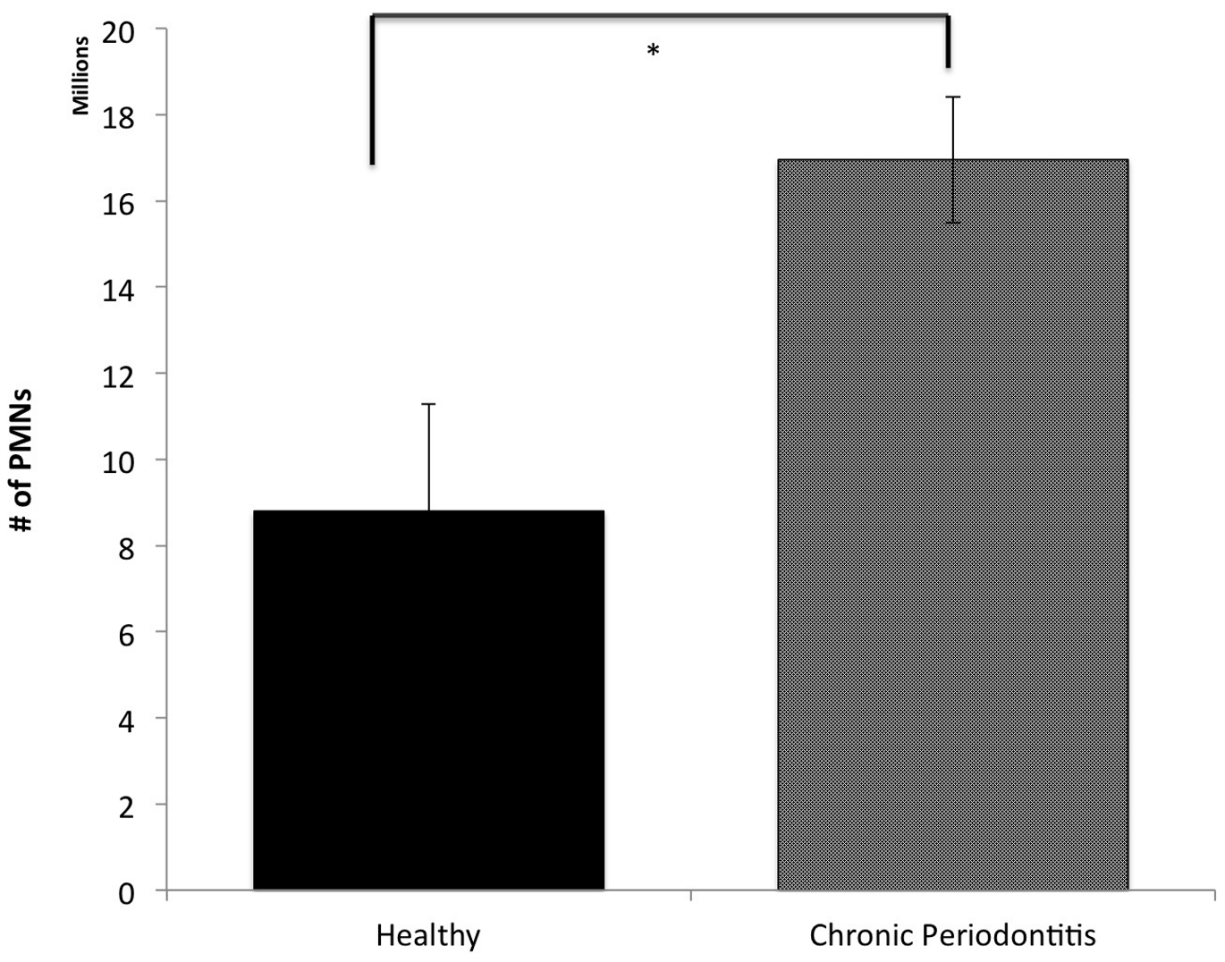
Oral chronic inflammation increases neutrophil recruitment to the site of infection. The proportion of neutrophils recruited to the oral cavity was counted in healthy patients, patients with chronic periodontits. * p ≤ 0.02; vs. Healthy. All data are mean ± SEM. Healthy subjects (n = 14); Generalized Periodontitis (n = 62).

We were interested in comparing the number of neutrophils recruited to the oral cavity in healthy subjects and in patients with CP as we have previously shown that oral inflammation correlates with oral neutrophil levels [9]. Patients with CP presented with more than a 2.5 fold increase in mean oral neutrophils compared to healthy subjects (p-value ≤0.02; Figure 1). Although the age difference between healthy subjects and CP patients is statistically significant, according to the National Health and Nutrition Examination Survey, 1999–2004 of the United States, the total age range in both groups belong to the same group risk to develop periodontal disease. Where 12% of all patients age 50 to 64 years have periodontal disease [33].

### Neutrophils Alter their Gene Expression Profile as they Transit from Circulation into the Oral Tissues

**Figure 2:**
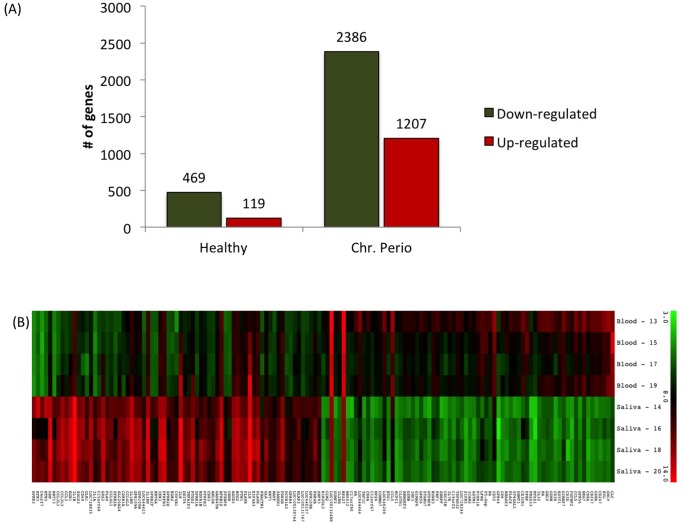
The oral neutrophil in chronic periodontitis has high transcriptional activity compared to the oral neutrophil in a healthy patient. (A) Graphic representation of the number of genes that are either up regulated (red) or down regulated (green), when blood neutrophils (PMN-B) were compared with oral neutrophils (PMN-O) in healthy individuals and chronic periodontitis patients FC ≥2; p-value ≤0.05. (B) Heatmap of genes with FC ≥5. Genes shown in red are up-regulated and those shown in green are down-regulated in Oral samples of Chronic Periodontitis patients (n = 4). The complete list of genes can be found at the file S1.

We used whole genome expression to compare neutrophils from blood (PMN-B) and oral rinses (PMN-O) from healthy subjects and patients with CP. Microarray analysis revealed that neutrophils alter their transcriptome as they transit from circulation into the oral cavity in both healthy and CP groups. However, changes in neutrophil gene expression, as measured by numbers of genes either up regulated or down regulated, was more extensive in the disease group. Of the 47,231 probe sets in the Illumina chip, 588 genes were differentially expressed between PMN-B and PMN-O in healthy subjects while 3,593 genes were differentially expressed between PMN-B and PMN-O in the CP patients (cut-off values for gene analysis was 2X fold change and with a p-value of 0.05) (Figure 2 - A). The extent of this change is visualized in a heat map of genes selected by a fold change (FC) of 5 or higher (Figure 1 - B). A list of top 50 differentially expressed genes in PMN-O between Healthy and CP patients can be found in File S1. The complete list of the differentially regulated genes can be viewed online at GEO website (http://www.ncbi.nlm.nih.gov/geo/) under accession number GSE 435525.

### Commonly Regulated Functions in Healthy Subjects and Chronic Periodontitis Patients

After identifying the differentially expressed genes from our microarray experiment, we used IPA software, DAVID 6.7 (http://david.abcc.ncifcrf.gov) (the database for annotation, visualization and integrated discovery) and the GOEAST (http://omicslab.genetics.ac.cn/GOEAST/) (Gene Ontology Enrichment Analysis Software Toolkit) bioinformatics tools for post-analysis. Initial screening by the IPA software revealed differences in the expression levels of several genes that were clustered in specific categories. Using this bioinformatics approach, we identified key biologic processes that were involved as neutrophils leave circulation and enter a site of inflammation. Among the physiological functions that were enriched both in healthy and CP patients we found Hematological System Development and Function, Tissue Development and Immune Cell Trafficking to be up regulated in oral neutrophils (File S2). Additional analysis was performed using DAVID in order to organize differentially regulated genes into canonical pathways. Among the canonical pathways in common in healthy subjects and CP patients, when comparing PMN-B vs. PMN-O we found Cytokine-cytokine receptor interaction, Chemokine signaling pathway, Hematopoietic cell lineage, T cell activation and Inflammation mediated by chemokine and cytokine signaling pathway (Table 3 and File S3).

**Table 3:** DAVID pathway analysis – Common pathways of Chronic Periodontitis and Healthy Subjects.

Pathway iD	Term
hsa03010	Ribosome
hsa05332	Graft-versus-host disease
hsa05340	Primary immunodeficiency
hsa04060	Cytokine-cytokine receptor interaction
hsa04640	Hematopoietic cell lineage
hsa04062	Chemokine signaling pathway
hsa04650	Natural killer cell mediated cytotoxicity
hsa05020	Prion diseases
h_tcraPathway	Lck and Fyn tyrosine kinases in initiation of TCR Activation
h_tcrPathway	T Cell Receptor Signaling Pathway
h_tcytotoxicPathway	T Cytotoxic Cell Surface Molecules
h_ctlPathway	CTL mediated immune response against target cells
h_setPathway	Granzyme A mediated Apoptosis Pathway
h_lympathway	Adhesion and Diapedesis of Lymphocytes
REACT_1762	3′ -UTR-mediated translational regulation
REACT_6167	Influenza Infection
REACT_17015	Metabolism of proteins
REACT_71	Gene Expression
REACT_6900	Signaling in Immune system
P00031	Inflammation mediated by chemokine and cytokine signaling pathway
P00053	T cell activation

Additional analysis can be found in File S3.

**Figure 3:**
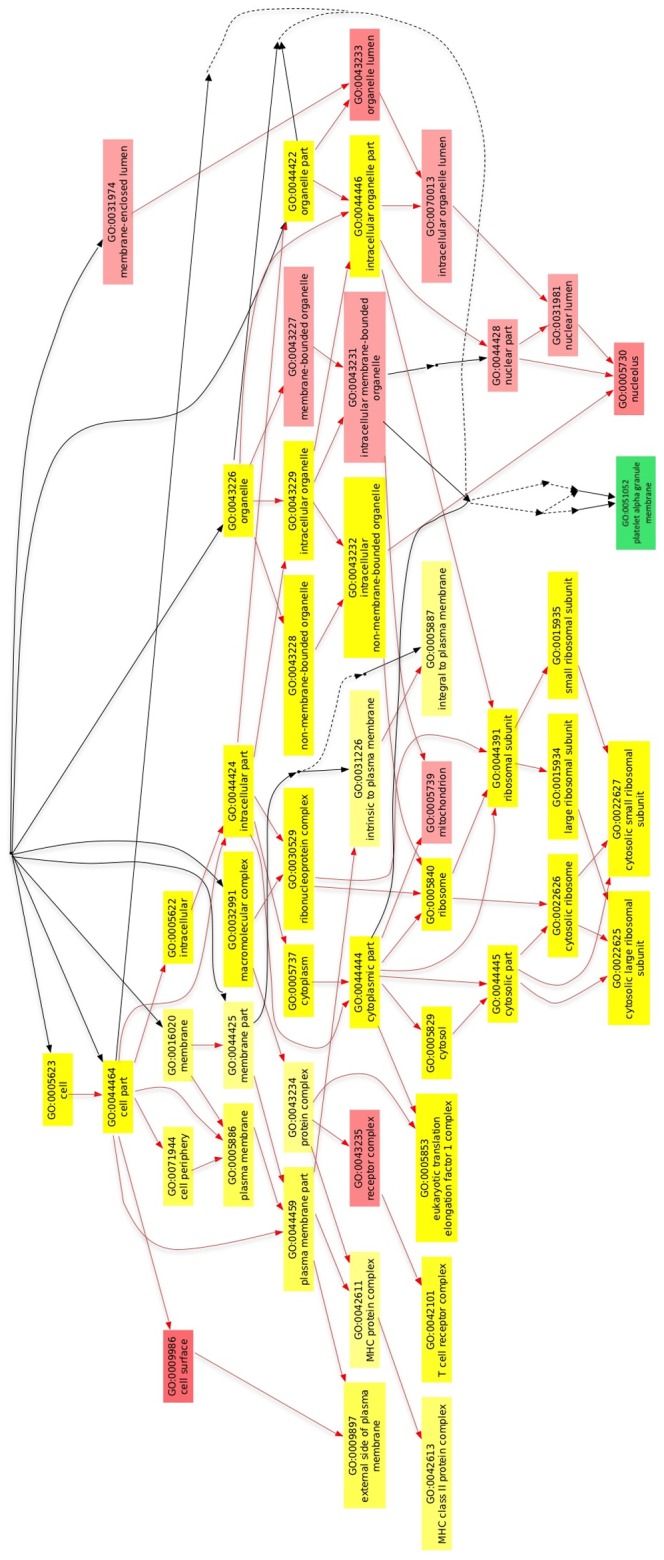
Multi analysis of Gene Ontology (GO) annotations demonstrates that some key functions are preserved during inflammatory process in the mouth. The GO terms grouped into Cellular Components that are uniquely enriched in healthy patients (green) and chronic periodontits patients (red). Significantly enriched GO terms in both comparisons are marked yellow. The degree of color saturation of each node is positively correlated with the significance of enrichment of the corresponding GO term. Red arrows stand for relationship between two enriched GO terms, black solid arrows stand for relationship between enriched and unenriched terms.

Using the Multi-GOEAST, we compared Gene ontology (GO) terms that were used for enrichment analysis of Healthy subjects and CP patients. As demonstrated in Figure 3, after selecting for Cellular Component, several GO categories were similarly enriched in CP patients when compared with healthy subjects, demonstrating that key cellular functions are preserved in a diseased state (Figure 3). Additional analysis regarding Molecular function and Biological Function are provided in File S4 and follows the same trend.

### Differently Regulated Functions in Healthy Subjects and CP Patients

Among the pathways that were regulated in CP patients only, which were grouped into the apoptosis category using DAVID cluster analysis, we observed up-regulation of Toll-like receptor signaling pathway, Caspase Cascade in Apoptosis, Apoptosis signaling pathway and Cell Cycle Checkpoints (Table 4 and File S4). We found nucleotide-binding oligomerization domain (NOD-like) receptor signaling pathway, Cell adhesion molecules (CAMs) and Interleukin 17 (IL 17) Signaling Pathway were uniquely enriched in Healthy subjects.

**Table 4:** DAVID pathway analysis –Pathways up-regulated in Chronic Periodontitis patients only.

Pathway iD	Term
hsa03040	Spliceosome
hsa04620	Toll-like receptor signaling pathway
hsa04142	Lysosome
hsa04650	Natural killer cell mediated cytotoxicity
hsa05010	Alzheimer’s disease
hsa04144	Endocytosis
hsa05012	Parkinson’s disease
h_WNVpathway	West Nile Virus
h_caspasePathway	Caspase Cascade in Apoptosis
h_ptdinsPathway	Phosphoinositides and their downstream targets.
REACT_6185	HIV Infection
REACT_383	DNA Replication
REACT_578	Apoptosis
REACT_13635	Regulation of activated PAK-2p34 by proteasome mediated degradation
REACT_1538	Cell Cycle Checkpoints
REACT_9035	APC/CCdh1-mediated degradation of Skp2
REACT_11045	Signaling by Wnt
REACT_6850	Cdc20Phospho-APC/C mediated degradation of Cyclin A
P00049	Parkinson disease
P00006	Apoptosis signaling pathway

Additional analysis can be found in File S3.

### Apoptosis Associated Networks are Significantly Influenced by Chronic inflammation in the Oral Cavity

**Figure 4:**
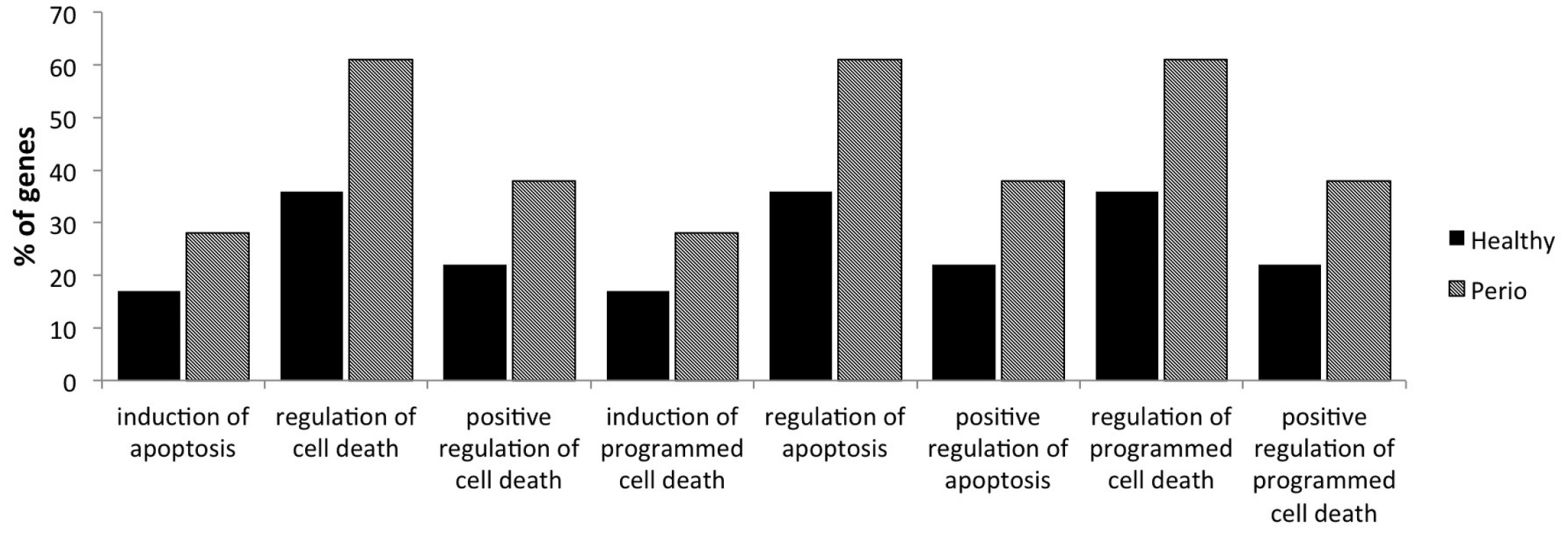
Cluster analysis of Gene Ontology (GO) annotations reveals apoptosis regulation as one of the main changes in the neutrophil transcriptome pattern in an inflamed site. The GO terms assigned to healthy patients (black) and chronic periodontits patients (striped) were grouped into clusters. In general, a similar percentage of genes were found in most categories. The apparently significant difference between the number of genes from healthy patients and chronic periodontitis patients falls into the apoptosis cluster. Differences in demonstrated categories are significant.

**Figure 5:**
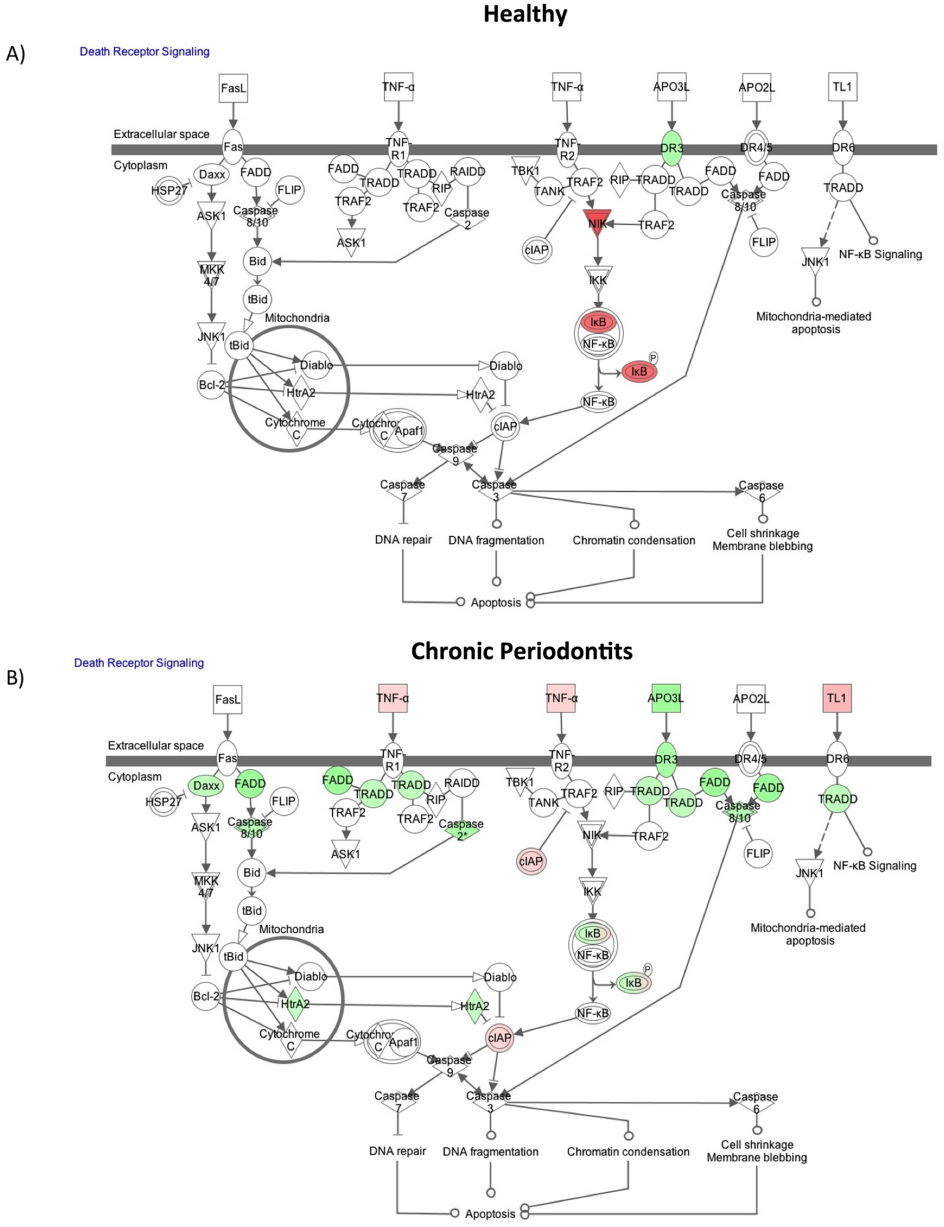
Graphic representation of Death Receptor Pathway and Apoptosis Signaling Pathway in neutrophils demonstrates that they are altered in neutrophils in chronic inflammation. IPA canonical pathway analysis of Death Receptor Signaling Pathway in neutrophils from (A) healthy individuals and (B) chronic periodontitis patients. Red represents up-regulated genes, green are down-regulated genes and white symbols depict neighbouring genes in this analysis.

Next we used the cluster analysis function of DAVID, that arranges genes according to similarity in pattern of gene expression [28]. These clusters fell within each of the larger ontologies of: Cell activation, apoptosis and metabolic processes. The ontology groups with significant difference between the number of genes from healthy patients and CP patients falls into the apoptosis, cell activation and cellular component clusters (Figure 4–5).

### Functional Confirmation of Microarray Data with qRT-PCR, Western Blotting and by Flow Cytometry

**Figure 6:**
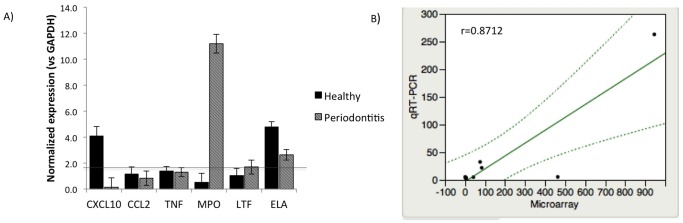
Validation of microarray data with qRT-PCR. (A) Quantitative real time PCR was used to quantify gene expression of selected genes from neutrophils isolates from blood and oral rinse. Results are expressed as fold vs. Blood expression used as internal control. Gene expression was normalized with glyceraldehyde-3-phosphate dehydrogenase (GAPDH) as a reference gene. (B) Correspondence between mean fold change (FC) values obtained by microarray (X) vs. qRT-PCR (y) analysis. The diagonal line represents the ideal correspondence trend (Pearson correlation r = 0.8712, p-value ≤0.01). All data are mean ± SEM from 3 independent experiments (n = 4).

We used qRT-PCR experiments to confirm microarray results from PMN-B and PMN-O in healthy subjects and CP patients by correlating the signal intensities of the microarray with those from qRT-PCR of selected genes. Selected genes, identified by the microarray analysis, were analyzed using quantitative RT-PCR (qRT-PCR). The selected genes represented two groups: the first represented neutrophil-specific genes [myeloperoxidase (MPO), neutrophil lactoferrin (LTF), neutrophil expressed elastase (ELA)] and the second, genes involved in the overall inflammatory response [chemokine (C-X-C motif) ligand 10 (CXCL10), chemokine (C-C motif) ligand 2 (CCL2), tumor necrosis factor (TNF)]. Pearson linear correlation test was used to measure the relationship between the qRT-PCR and microarray experiments (Pearson correlation r = 0.8712, p-value ≤0.01). The qRT-PCR confirmed the data generated through microarray. In general the results followed the same trend with differences in the magnitude of these changes (Figure S1). The gene expression levels of selected genes from neutrophil isolates from blood and oral rinse were measured by real-time PCR (Figure 6).

**Figure 7:**
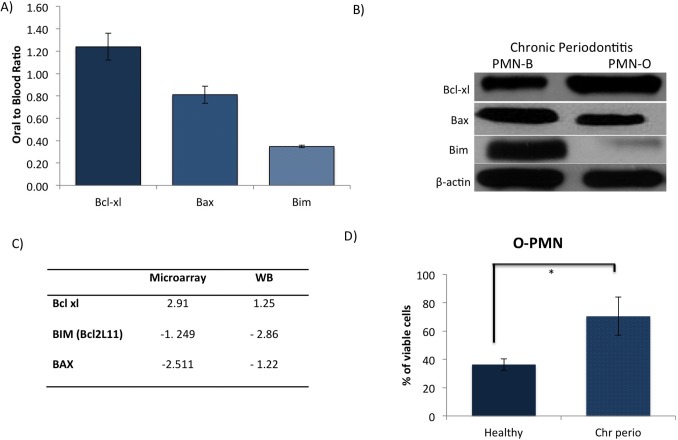
Functional confirmation of microarray data with Western Blotting and by Flow Cytometry – Presenting up-regulation of anti-apoptosis and down-regulation of pro-apoptosis proteins. (A) Western blotting quantification; 15 µg of protein from total cell lysate of PMN-B and PMN-O from chronic periodontitis patients were loaded, expression was normalized to β-actin used as internal control (n = 4). (B) Representative Western Blot of members of apoptosis pathway Bcl-xl (anti-apoptosis), Bim and Bax (pro-apoptosis). (C) Summary of relative changes in BCL-2 family (Ratio of expression in PMN-O compared to PMN-B). (D) Percentages of viable cells were analyzed by Flow cytometry in oral neutrophils (n = 3). Cells that were negative for Annexin V and PI were considered viable. * p ≤ 0.05 vs. Healthy. All data are mean ± SEM from 3 independent experiments.

After having demonstrated the expression of a variety of Bcl-2-related transcripts in oral neutrophils from CP patients, we wanted to confirm the expression of these genes at the protein level. Using Western Blot analysis we quantified protein expression of Bcl-2 (Bcl-xl), a pro-survival member of the mammalian Bcl-2 family of apoptosis-associated proteins, and Bax, a member of Bcl-2 family, that is known to induce apoptosis (Figure 7– A, B). We found that oral neutrophils from CP patients present increased expression of Bcl-2 and reduced expression of Bax. This matched what we found in the gene expression analyses. At protein level Bax expression follows the same trend with 1.22 fold decrease in PMN-O (p-value = 0.05). Bcl-2 expression (FC = 1.25; p-value = 0.06) is up-regulated in O-PMN of CP patients. We also found Bim (Bcl2L11), another pro-apoptosis member of Bcl-2 family, to be significantly down regulated in oral neutrophils of CP patients (FC = −2.86; p-value = 0.01). A summary of the relative changes in the Bcl-2 family can be found in figure 7 - C.

Finally, using flow cytometric analysis we measured cell viability in oral neutrophils from CP and healthy patients. We found that oral neutrophils from CP patients present increased viability (70±13.43%), when we compared with healthy subjects (36±7.67%; p-value ≤0.05) and decreased necrotic events (4±1.14% vs. 41±8.73%; p-value≤0.02) (Figure 7 - D). Similar results were described in the literature, where oral neutrophils death is by necrosis rather than by apoptosis [34].

## Discussion

The purpose of the present study was to compare the gene expression profile changes in neutrophils as they enter the oral tissues of patients with periodontal diseases and healthy subjects. We identified the gene expression changes in the oral neutrophils of CP patients and observed that in addition to the regulation of genes in the Toll-like receptor (TLR) signaling pathway and other inflammation pathways a significant number of genes were altered with the apoptosis regulatory pathway being highly significant. This was of significance to us since it has been suggested that neutrophil numbers regulate the magnitude and length of the inflammatory response [11], [35]. Longer lived neutrophils may result in a more severe inflammatory response and more periodontal tissue destruction.

### Understanding Neutrophil Biology

Many studies have used microarray to investigate differences in the transcriptome of periodontal tissue cells (gingival biopsies) or circulating neutrophils from CP patients [17], [18], [20]. However, the present study is the first to focus on transcriptome changes in neutrophils as they migrate from circulation into the periodontal tissues. This data demonstrates that neutrophils undergo changes in their transcriptome as they move between circulation and tissues compartments. Moreover, these results show that these gene changes are dramatically increased in cases of severe inflammation compared to neutrophils that enter the healthy periodontium. This approach using methods to isolate pure tissue neutrophils combined with gene expression analysis allowed us to better understand the neutrophil and its changes once it leaves circulation in inflammatory diseases.

In addition to the protective role in oral health maintenance, neutrophils have been shown to play an important role in the pathogenesis and progression of periodontitis. Several neutrophil mediated mechanisms have been suggested to contribute to periodontal tissue breakdown: 1) the release of proteases such as collagenase and elastase which leads to damage of the connective tissues and 2) reactive oxygen species (ROS) release that triggers oxidative stress and DNA damage responses, leading to irreversible cell membrane damage and induction of apoptosis [8], [36]–[38].

Until recently the proposed role of neutrophils in periodontal disease were linked to impaired function [39], [40] or to hyperactive phenotype [21], [41], [42]. A third contributory category was described with a increased recruitment [9], and activation of the normal neutrophil [36], [43], [44] and also with decreased apoptosis of neutrophils, which leads to longer life span of the neutrophil in the site [44], [45]. Here, we could demonstrate that neutrophils in the oral cavity of CP patients present a transcriptome that is characterized by an increased expression of chemokines, IL-1 members and regulators of apoptotic/cell death events.

### Mediators of Neutrophil Recruitment

Consistent with this increase in neutrophils in the oral environment we observed up-regulation of chemokine (C-C motif) ligand 3 [CCL3 (MIP-1α)], a CC chemokines, that is recognized as a potent chemoattractant for neutrophils [45]. CCL3 was also described in the context of resolution of inflammation, where its down-regulation regulates a later resolution phase [46]. Increased expression levels of CCL3 in PMN-O from CP patients could be also linked to the amplification of neutrophil recruitment to the mouth, similar to what was reported in the synovial fluid from patients with rheumatoid arthritis [47]. We also noted up regulation of Interleukin 1 (IL-1) which is known to act as a regulator of neutrophil recruitment in the periodontal tissue as described in a previous work from Graves’ group [48]. They were able to demonstrate the link between IL-1 activity and the pathologic process of periodontitis, where inhibition of IL-1 reduced the recruitment of inflammatory cells in close proximity to the bone, resulting in a 60% reduction in bone loss [48]. Consistent with this, it has also been shown, in vitro, that IL-1 induces osteoclast bone resorption [49].

### Oral Neutrophil Survival

Since our data showed that genes responsible for neutrophil survival were differently regulated, we confirmed by a functional assay that oral neutrophils from CP patients displayed increased viability. Demmer *et al.* [18] using microarray identified pathways that were differentially expressed in diseased and healthy periodontal tissue. Similar to our findings, he found that induction of apoptosis was altered, but unlike our study, he did not verify by q-PCR and Western blotting which specific components were altered. In addition, they did not assess which cells were responsible for these gene changes as they were looking at all cells in the tissue biopsies. Catalase was among the genes that we found to be down-regulated in PMN-O from CP patients that wasn’t reported in Demmer’s study, downstream products of this pathway play a crucial role in cellular anti-oxidant defense and the resolution of inflammation (File S1) [50].

It is interesting to note that although we found that cell survival is increased, some key regulators of the apoptosis pathway were identified in the hierarchical cluster analysis as belonging to the category of regulators of apoptosis and cell survival. These include Interleukin 1 beta (IL-1β) (a potent anti-apoptotic molecule [51], [52]), and Interleukin 6 (IL-6). Similar findings of higher expression of IL-6 in periodontal tissues from chronic periodontitis were recently reported [53]. IL-6 is linked to generation and maintenance of chronic inflammation, where pro-inflammatory cytokines stimulate the release of IL-6 by neutrophils [54]. IL-6 release would increase the expression of adhesion molecules such as vascular cell adhesion molecule 1 (VCAM-1) and Intercellular Adhesion Molecule 1 (ICAM-1), promoting neutrophil accumulation in the tissue [54]. Notably and consistent with our study IL-6 has been linked to an anti-apoptotic effect in neutrophils [55], [56].

Consistent with our findings, the TLR pathway that was found to be up-regulated in oral neutrophils of CP patients. Up-regulation of the TLR pathway is linked to the pathogenesis of a number of chronic diseases such as rheumatoid arthritis [57], chronic asthma [58] and sepsis [59] indicating a hyperactive neutrophil phenotype [60]. Recently, Chakravarti *et al.* [61] demonstrated that neutrophils up-regulate their expression of membrane Receptor activator of nuclear factor kappa-B ligand (RANKL) after lipopolysaccharide (LPS) stimulation through TLR2 and TLR4, thereby implicating these genes in activating osteoclastic bone resorption in RA patients [61]. This is likely to lead to a similar mechanism of neutrophil initiated periodontal bone loss. There is also compelling evidence showing that TLR members can mediate intracellular signaling to modulate Bcl-2 proteins, leading to increased cell survival [62], [63].

In summary, consistent with what was described by Coldren *et al.* [64], who used an in vivo-pulmonary neutrophil transmigration model in humans [64], we found altered regulation of the apoptosis pathway, that results in increased viability of neutrophils in the inflamed oral tissues. Furthermore, we have identified differentially expressed genes in oral neutrophils from patients with periodontal disease that are not expressed in oral neutrophils from healthy patients. This data could potentially be used to identify biomarkers of periodontal disease activity, which could help overcome of challenge of identifying patients in the active phase of this inflammatory disease process.

## Supporting Information

Figure S1 **Validation of microarray data with qRT-PCR.** Verification of microarray data by qRT-PCR. Results are expressed as the average relative fold-change in transcripts from PMN-O compared to PMN-B expression. Results are expressed as fold vs. Blood expression used as internal control and qRT-PCR analysis was normalized with GAPDH as a reference gene, as described in Methods.
(TIFF)

File S1 List of top 50 differentially expressed genes in PMN-O between Healthy and CP patients.
(XLSX)

File S2 Summary of IPA analysis - Humans - Blood vs. Oral PMNs (Chronic Periodontitis Patients).
(XLSX)

File S3 DAVID Pathway analysis-Healthy patients - Blood vs. Oral PMNs.
(XLSX)

File S4 Multi-GOEAST analysis -Chr. Periodontitis & Healthy - Blood vs. Oral PMNs.
(XLSX)
