# Supplementary figures and images for: Oral Neutrophil Transcriptome Changes Result in a Pro-Survival Phenotype in Periodontal Diseases
Source: PLoS One. 2013 Jul 11;8(7):e68983. doi: 10.1371/journal.pone.0068983 (PMC3708893; doi:10.1371/journal.pone.0068983)

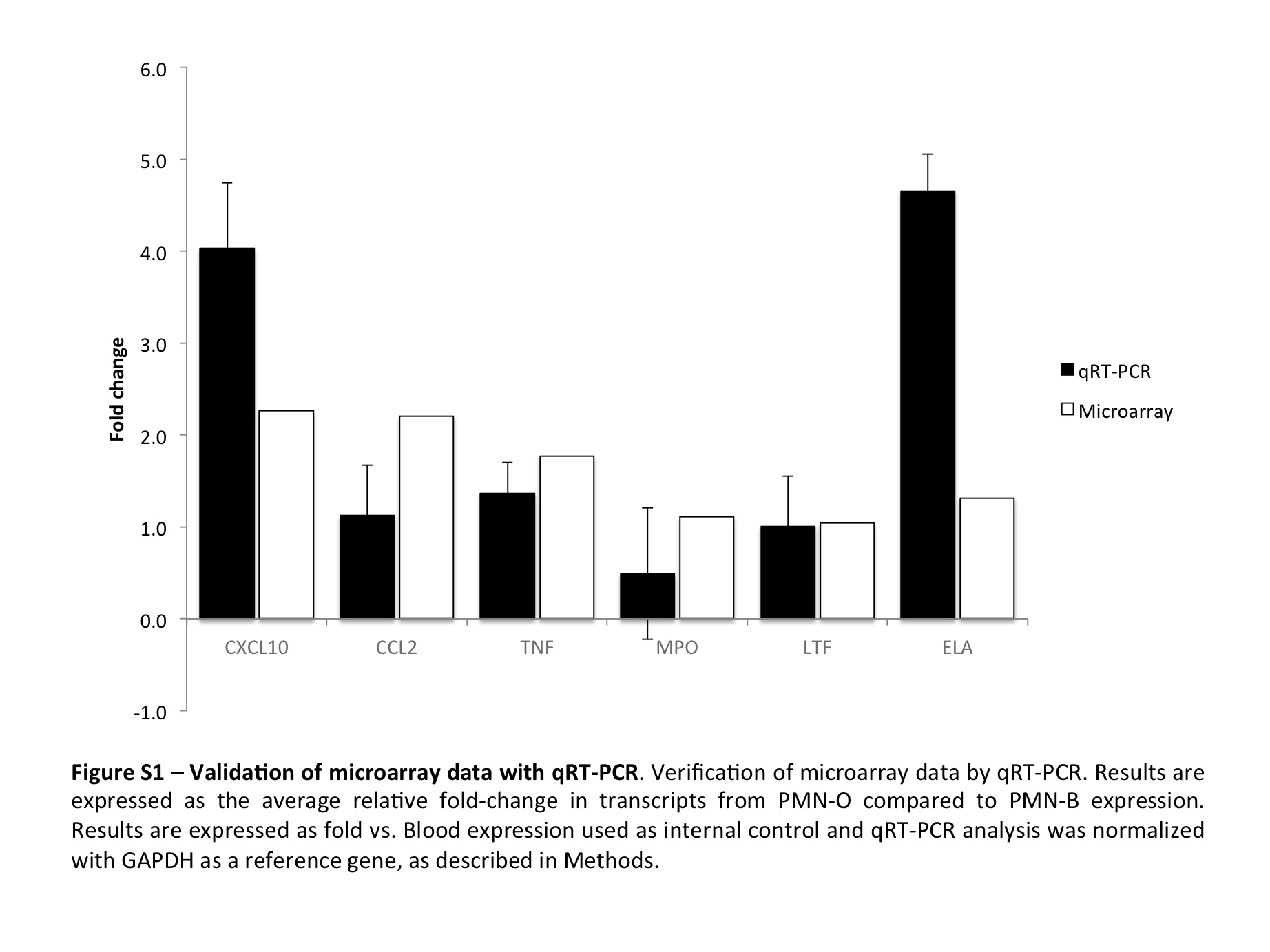

Supplement: Figure S1 — Validation of microarray data with qRT-PCR. Verification of microarray data by qRT-PCR. Results are expressed as the average relative fold-change in transcripts from PMN-O compared to PMN-B expression. Results are expressed as fold vs. Blood expression used as internal control and qRT-PCR analysis was normalized with GAPDH as a reference gene, as described in Methods. (TIFF) [file pone.0068983.s001.tiff]
